# Case report: The value of early application of mNGS technology in the diagnosis and treatment of severe Legionnaires’ disease: reports of two cases with different outcomes

**DOI:** 10.3389/fmed.2025.1501192

**Published:** 2025-02-05

**Authors:** Jianqing Fang, Zhe Wang, Yu Shen, Xuenong Wu, Hao Fang, Xiaokui Sun, Ting Yu, Qingqing Zhang

**Affiliations:** ^1^Department of Respiratory and Critical Care Medicine, Hangzhou Linping Hospital of Traditional Chinese Medicine, Hangzhou, China; ^2^Department of Ultrasound Medicine, Hangzhou Linping Hospital of Traditional Chinese Medicine, Hangzhou, China; ^3^Department of Intensive Care Medicine, Hangzhou Linping Hospital of Traditional Chinese Medicine, Hangzhou, China

**Keywords:** legionellosis, Legionnaires’ disease, case report, severe community-acquired pneumonia, severe hospital-acquired pneumonia

## Abstract

**Background:**

Legionnaires’ disease has a high clinical mortality rate, and early diagnosis and treatment are critical. Increasing evidence shows that metagenomic next-generation sequencing (mNGS) has excellent potential for the early identification of pathogens. To help clinicians better recognize Legionnaires’ disease in its early stage and to illustrate the diagnostic value of mNGS technology, we reviewed and summarized two cases of severe Legionnaires’ disease.

**Methods and analysis:**

We selected two patients with severe Legionnaires’ disease who were admitted to our department in recent years. We discuss experience with them and the shortcomings in their treatment by summarizing their medical history, disease evolution, tests, and diagnostic and therapeutic processes.

**Results:**

In both patients, the diagnosis of Legionnaires’ disease was confirmed through analysis of the bronchoalveolar lavage fluid (BALF). The middle-aged male patient was cured and discharged due to early detection and diagnosis. The elderly immunocompromised patient died due to a delay in diagnosis.

**Conclusion:**

This study highlights the importance of the early recognition and diagnosis of severe Legionnaires’ disease and the advantages of mNGS in identifying the pathogen.

## Introduction

*Legionella* spp. are intracellular-parasitic, aerobic, gram-negative bacilli, of which 58 species and more than 70 serotypes have been identified (1, 2). These pathogens were first recognized following an outbreak of Legionnaires’ disease (LD) at the 1976 American Legion Convention in Philadelphia (3). As a conditionally pathogenic bacterium, *Legionella* is widespread in natural water systems, soil, air-conditioning systems, and water catchment systems (4, 5). *Legionella* is spread mainly by inhalation of bacterial aerosols arising from contaminated water or soil (1).

*Legionella* infection can present as mild, self-limiting, flu-like symptoms; this condition is known as Pontiac fever and usually does not require antimicrobial treatment (2, 6). Patients with high-risk factors for infection, including chronic lung disease, age > 50 years, glucocorticoid therapy, hematological malignancies or solid tumors, and organ transplantation (7), are prone to opportunistic infection leading to community-acquired or hospital-acquired LD (1). Smoking can also create an environment that is conducive to the proliferation of microbes within the bronchial tree (8). A previous study has demonstrated that smoking is the most significant risk factor in LD patients (9). LD can progress to severe pneumonia or even severe acute respiratory distress syndrome, requiring intensive care and extracorporeal membrane oxygenation (ECMO) treatment (10). In immunocompromised patients, *Legionella* can also cause extrapulmonary legionellosis through blood dissemination (11), such as pericarditis (12), endocarditis (13), meningitis (14), and liver infection (15). *Legionella pneumophila* (Lp), *Legionella micdadei*, *Legionella longbeachae*, *Legionella bozemanae*, and *Legionella dumoffii* are the species that are most commonly encountered clinically. Among all *Legionella* spp., *Legionella pneumophila* serogroup 1 (Lp1) is the most virulent and common causative agent of LD (16); approximately 90% of LD cases result from Lp1 infection (17, 18).

The clinical manifestations of LD vary and include chills, fever, cough, hemoptysis, general malaise, and relative bradypnea. Gastrointestinal and neurological symptoms such as diarrhea, nausea or vomiting, and headache may be more prominent than they are in other bacterial pneumonias (2). Laboratory tests of LD patients have revealed leukocytosis with relative lymphopenia and elevated C-reactive protein (CRP) and calcitoninogen levels. Hypernatremia, hypophosphatemia, elevated levels of liver enzymes, and creatine kinase are common in this disease (19). The imaging presentation lacks specificity; early lesions are located mainly in unilateral lobes of the lungs and present as patchy or interstitial exudative opacity with blurred borders; this appearance can rapidly progress to consolidation in the short term, and pleural effusions and necrotic cavities are sometimes observed (20). In immunosuppressed patients, extrapulmonary dissemination and recurrence are more likely, and pulmonary cavitation is also more common, resulting in a higher mortality rate (21).

Because *Legionella* parasitizes alveolar macrophages, antibiotics that do not penetrate the host cell membrane, such as common beta-lactams and aminoglycosides, are ineffective. The antibiotics available for treatment include fluoroquinolones, macrolides, and rifampicin. Fluoroquinolone or macrolide monotherapy for LD, typically involving the use of levofloxacin, moxifloxacin, azithromycin, or clarithromycin, is still the conventional regimen (22–25). In patients with severe pneumonia, especially those with severe comorbidities and immunocompromised patients who have failed to respond to monotherapy regimens, fluoroquinolones in combination with macrolides may be considered (26). *Legionella* remains susceptible to commonly used antibiotics, and reports of resistance are rare (27, 28). This article reports two typical cases of severe LD with different outcomes.

## Case 1 presentation

**FIGURE 1:**
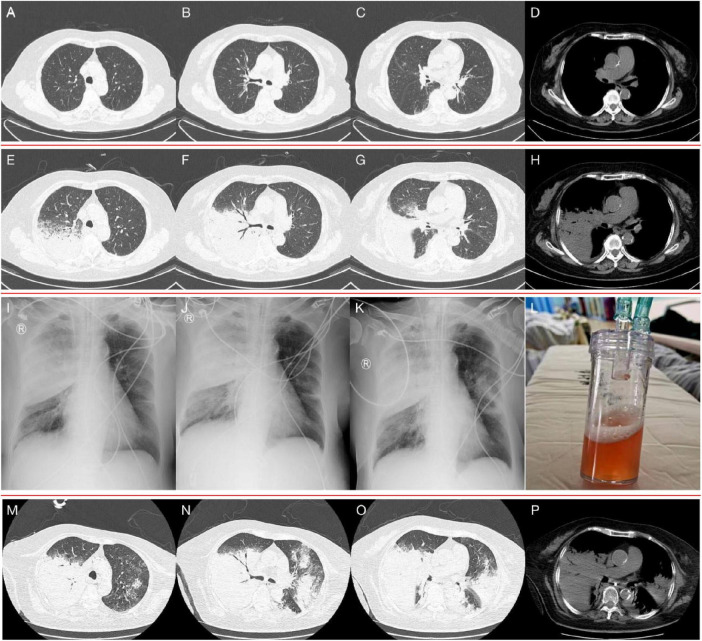
**(A–D)** Chest CT on admission, showed emphysema and slight thickening of the bronchial wall. **(E–H)** Chest CT on hospital day 16, showed a new sizeable consolidation in the upper right lung with blurred borders, a bronchial inflation sign, slightly patchy exudative opacity in the right middle lobe, and a small amount of pleural effusion on the right chest. **(I–K)** Bedside chest radiography on hospital day 18, 20, and 22, respectively, showed gradual enlargement of consolidation in the upper right lung and the development of new patchy exudative opacity in the upper left lung. **(L)** orange-colored BALF was collected at RB2 on hospital day 22. **(M–P)** Chest CT on hospital day 24, showed significant progression of the lesions and exudative consolidation in multiple lobes of both lungs.

An 80-year-old woman with a 5-day history of cough and shortness of breath was admitted to the respiratory department during the summer months. She had a medical history of chronic obstructive pulmonary disease (COPD), hypertension, and previous cerebral infarction and was regularly treated with a tiotropium powder inhaler, irbesartan dispersible tablets, and atorvastatin tablets. She had no history of smoking. Physical examination revealed a body temperature of 37.8°C, a blood pressure of 152/62 mmHg, a heart rate of 84 beats/minute, and a respiration rate of 20 breaths/minute. She was conscious, with a barrel chest. Bibasilar wheezing was detected on lung auscultation. Both lower limbs exhibited mild edema. The remaining examination results were unremarkable. Laboratory investigations revealed a white blood cell (WBC) count of 12.65 × 10^9^/L, an absolute neutrophil count (ANC) of 10.12 × 10^9^/L and a platelet (PLT) count of 153 × 10^9^/L. The serum CRP level was 75 mg/L, and the serum PCT level was 0.53 ng/ml. Arterial blood gas (ABG) analysis revealed an FiO2 of 29.00%, pH of 7.39, pCO_2_ of 62.1 mmHg, and pO_2_ of 68.7 mmHg (oxygenation index of 237 mmHg). Serum IgM antibodies against atypical pathogens (including *Mycoplasma pneumoniae*, *Chlamydia pneumoniae* and LP), upper respiratory tract specimens PCR (including influenza virus, adenovirus and SARS-CoV-2), and blood and sputum cultures were all negative. Chest computed tomography (CT) on admission revealed emphysema and slight thickening of the bronchial wall (Figures 1A–D).

**FIGURE 2:**
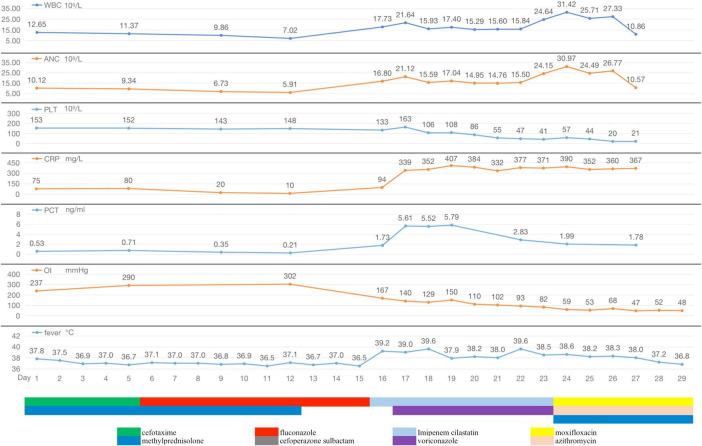
During hospitalization, the changes of body temperature, oxygenation index (OI), inflammatory indicators (WBC, ANC, PLT, CRP, and PCT), and antibiotic regimen adjustment process of Case 1.

Diagnosis and treatment: The patient’s initial diagnosis was acute exacerbation of chronic obstructive pulmonary disease (AECOPD). The treatment regimen included cefotaxime (2.0 g, ivgtt, q12h) as an anti-infective, methylprednisolone (20 mg, iv, qd) and nebulized inhalation (budesonide 2 mg plus terbutaline 5 mg, bid) to relieve airway spasms and the use of a non-invasive ventilator (Philips V60) to improve ventilation. On hospital day 6, the patient’s temperature was normal, but she still experienced shortness of breath and had yellowish sputum. On physical examination, her oral mucosal leukoplakia was detected, and moist rales and phlegm sounds were heard on lung auscultation. Repeat laboratory tests revealed a WBC count of 11.37 × 10^9^/L, an ANC of 9.34 × 10^9^/L, and a PLT of 152 × 10^9^/L. Her CRP level had increased to 80 mg/L, her PCT level was 0.71 ng/ml, and sputum culture revealed *Candida albicans* (+ +). The antibiotic coverage was broadened to include cefoperazone sulbactam (2.0 g, ivgtt, q8h) and fluconazole (0.2 g, ivgtt, qd) to cover *Pseudomonas aeruginosa* and *Candida albicans*, and methylprednisolone was discontinued. After 7 days of treatment, the patient’s symptoms improved, her oral mucosal leukoplakia subsided, her CRP and PCT levels returned to normal, and a repeat sputum culture was negative. Cefoperazone sulbactam was discontinued, fluconazole administration was switched to capsules (0.2 g, po, qd), non-invasive ventilator therapy was continued, and the patient was prepared for elective discharge. On hospital day 16, the patient developed chills and a high fever (39.2°C), with worsening dyspnoea. Examination revealed bilateral moist rales and marked upper right lung sounds. Laboratory investigations revealed that the WBC count had increased to 17.73 × 10^9^/L, the CRP level was 94 mg/L, and the PCT level was 1.73 ng/ml. ABG analysis revealed an FiO2 of 33.00%, pH of 7.42, pCO2 of 40.3 mmHg, and pO2 of 55.1 mmHg (oxygenation index 167 mmHg). Repeat chest CT revealed a new sizeable consolidation with blurred borders in the upper right lung, a bronchial inflation sign, slightly patchy exudative opacity in the right middle lobe, and a small amount of pleural effusion in the right chest (Figures 1E–H). Imipenem cilastatin (0.5 g, ivgtt, q6h) was empirically used for G-bacilli therapy. The next morning, the patient’s respiratory status worsened, with persistent dyspnea during sitting, slightly blurred consciousness, and coughing up of pale blood-colored sputum. Many bibasilar crackles were heard on lung auscultation. Considering severe nosocomial pneumonia with acute heart failure (PSI score: 160 points, class V; NYHA: cardiac function class IV), we transferred her to the intensive care unit (ICU) for invasive mechanical ventilation. The ICU physician performed endotracheal suction and bedside rapid microscopic detection of fungal fluorescence; fungal spores were positive. Antibiotic therapy was escalated to imipenem cilastatin (0.5 g, ivgtt, q6h) and voriconazole (0.2 g, ivgtt, q12h) to cover G-bacilli and fungi. As her condition became unstable, the patient underwent bedside chest radiography every 2 days; this revealed gradual enlargement of the consolidation in the upper right lung and the development of new patchy exudative opacity in the upper left lung (Figures 1I–K). Her CRP and PCT levels did not improve greatly. Both blood and sputum cultures and serum anti-Lp IgM antibodies were negative. On hospital day 22, bronchoscopy was performed, and orange-colored bronchoalveolar lavage fluid (BALF) was collected at RB2 (Figure 1L). mNGS was performed using the PMseq platform, and the results were compared with those reported in the PMDB database. The mNGS results, which were obtained two days later, yielded 23,032 sequence reads for Lp (Table 1). The patient underwent chest CT, which revealed significant progression of the lesions and exudative consolidation in multiple lobes of both lungs (Figures 1M–P). On the basis of the patient’s clinical presentation and mNGS results, she was diagnosed with severe, nosocomial LD. The physician adjusted the antibiotic regimen by discontinuing imipenem cilastatin with voriconazole and starting her on moxifloxacin (0.4 g, ivgtt, qd) in combination with azithromycin (0.5 g, ivgtt, qd) for anti-*Legionella* therapy. Despite a series of comprehensive treatments, the patient’s condition continued to deteriorate, progressing to multiple organ failure (MOF), upper gastrointestinal bleeding, disseminated intravascular coagulation, and uncorrectable metabolic acidosis. The ICU physicians repeatedly recommended that the patient be transferred to a higher-level hospital for ECMO treatment, but the patient’s family refused. On hospital day 29, the patient’s family decided to abandon treatment. The patient died at home on the following day (Figure 2).

**TABLE 1:** mNGS results of the patient in case 1.

Genus name	Sequence number	Relative abundance%	Species name	Sequence number	Relative abundance%
*Legionella*	24392	99.95	*Legionella pneumophila* *Legionella anisa*	23032 23	94.38 /
*Candida*	3	/	*Candida tropicalis*	3	/
*Cutibacterium*	5	/	*Cutibacterium acnes*	3	/
*Human* *Alphaherpesvirus 1*	4	/			

## Case 2 presentation

**FIGURE 3:**
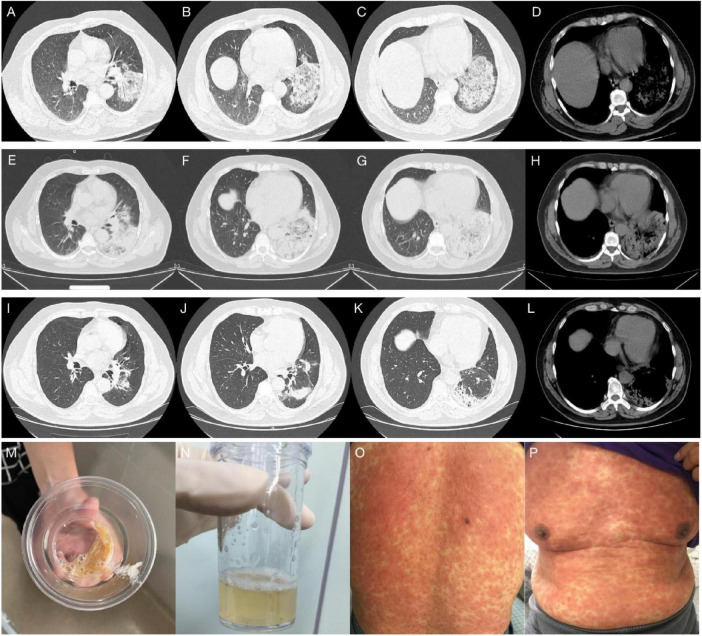
**(A–D)** Chest CT on admission, showed a large patchy exudative opacity with blurred borders and partial consolidation in the lower left lung, with thickening of the interlobular septum, a bronchial inflation sign and a small patch of exudative opacity in the lower lingual segment of the upper left lobe. **(E–H)** Chest CT on hospital day 4, showed rapid progression to consolidation in the whole lower left lung, accompanied by a small amount of bilateral pleural effusion. **(I–L)** Chest CT on hospital day 20, revealed significant absorption of consolidation in the left lung, leaving partial thickening of the left pleura and contraction of the interlobar fissure. **(M)** Pus-like, orange-yellow sputum. **(N)** orange-colored BALF was collected on LB10 on hospital day 2. **(O,P)** The patient suddenly developed a generalized maculopapular rash on hospital day 11.

A 50-year-old man who was a full-time taxi driver with no significant medical history was admitted to the respiratory department in early autumn, with a 3-day history of fever, cough and shortness of breath. His smoking index was 900. Physical examination revealed a body temperature of 40.4°C, a blood pressure of 108/63 mmHg, a heart rate of 126 beats/minute, and a respiration rate of 30 breaths/minute. He was conscious, with dyspnea and mild lip cyanosis. His lower left lung was turbid on percussion, and obvious moist rales were heard on auscultation. Laboratory investigations revealed a WBC of 17.88 × 10^9^/L, an ANC of 16.52 × 10^9^/L and a PLT of 137 × 10^9^/L. CRP was 273 mg/L, and PCT was 18.35 ng/ml. Serum chemical tests revealed an alanine aminotransferase (ALT) level of 104.3 U/L, an aspartate aminotransferase (AST) level of 350.8 U/L, an LDH level of 1099.2 U/L, a creatine kinase (CK) level of 8602.9 IU/L, a blood urea nitrogen (BUN) level of 11.40 mmol/L, sodium level of 133.1 mmol/L and an inorganic phosphorus level of 1.42 mmol/L. ABG analysis revealed an FiO2 of 33.00%, pH of 7.45, pCO2 of 31.2 mmHg, and pO2 of 61.9 mmHg (oxygenation index 188 mmHg). Serum IgM antibodies against atypical pathogens (including *Mycoplasma pneumoniae*, *Chlamydia pneumoniae*, and *Legionella pneumophila*), upper respiratory tract specimen PCR (including probes for influenza virus, adenovirus and SARS-CoV-2), blood and sputum cultures, and sputum for antacid bacilli were all negative. Chest CT on admission revealed a large patchy exudative opacity with blurred borders and partial consolidation in the lower left lung, with thickening of the interlobular septum, a bronchial inflation sign and a small patch of exudative opacity in the lower lingual segment of the upper left lobe (Figures 3A–D).

**FIGURE 4:**
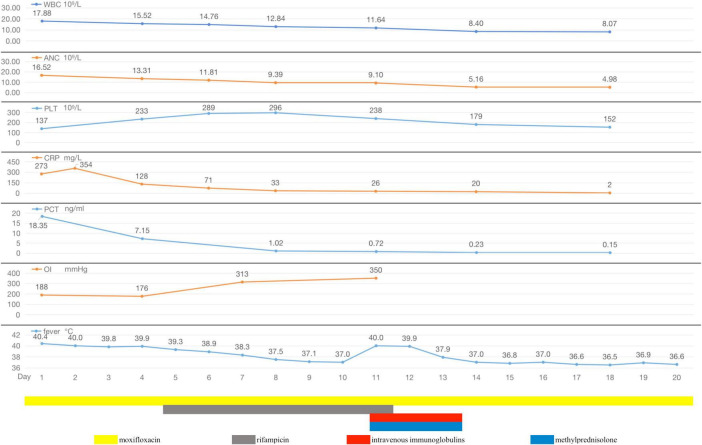
During hospitalization, the changes of body temperature, OI (oxygenation index), inflammatory indicators (WBC, ANC, PLT, CRP, and PCT), and antibiotic regimen adjustment process of Case 2.

The preliminary diagnosis was severe CAP (PSI score: 130, grade IV). As an empirical treatment regimen, moxifloxacin (0.4 g, ivgtt, qd) was given as an anti-infective agent against *Streptococcus pneumoniae* and atypical pathogens. After admission, the patient had pus-like, orange-yellow sputum (Figure 3M), but several sputum cultures were negative. Bronchoscopy under general anesthesia and endotracheal intubation were performed on hospital day 2, and orange-colored BALF was collected on LB10 (Figure 3N). The mNGS assay was performed using the PMseq platform; comparison of the results with the PMDB database, revealed 239 sequence readings for Lp 2 days later (Table 2). On the same day, repeat chest CT revealed rapid progression to consolidation in the entire lower left lung, accompanied by a small amount of bilateral pleural effusion (Figures 3E–H). The diagnosis of severe LD was confirmed on the basis of the patient’s history, clinical presentation, laboratory results, chest radiographic findings, and mNGS results. Given the patient’s severe condition at that time—he still had fever and dyspnea upon exertion, and a repeat ABG analysis revealed an oxygenation index of 176 mmHg—rifampicin (0.3 g, ivgtt, q12h) was added to his treatment. After 2 days of combination therapy, his fever subsided, and his clinical presentation improved markedly. However, on hospital day 11, the patient suddenly developed a generalized maculopapular rash (Figures 3O,P), without pain or itching, accompanied by a renewed high fever (40°C) without chills. The patient’s respiratory symptoms, such as cough and dyspnea, did not deteriorate at this time. Furthermore, his CRP and PCT decreased to 26 mg/L and 0.72 ng/mL, respectively. Therefore, it appeared likely that the rash was associated with Lp infection or with a reaction to rifampicin rather than with worsening of the LD. Rifampicin was discontinued, and moxifloxacin was continued, with the addition of methylprednisolone (40 mg, iv, qd) and intravenous immunoglobulins (IVIGs) (10 g, ivgtt, qd) to combat antihypersensitivity reactions. Three days later, the patient’s temperature returned to normal, and the rash gradually became lighter. Consequently, his methylprednisolone and IVIGs were discontinued, and his symptoms of fever and rash did not recur. On hospital day 20, the patient complained of only a slight dry cough with no other discomfort, and his CRP, PCT, liver function, renal function, electrolyte, and ABG results were all normal. Repeat chest CT revealed significant absorption of the consolidation in the left lung, resulting in partial thickening of the left pleura and contraction of the interlobar fissure (Figures 3I–L), and the patient was successfully discharged (Figure 4).

**TABLE 2:** mNGS results of the patient in case 2.

Genus name	Sequence number	Relative abundance%	Species name	Sequence number	Relative abundance%
*Legionella*	255	81.73	*Legionella pneumophila*	239	76.60
*Prevotella*	34	/	*Prevotella bivia*	11	/
			*Prevotella melaninogenica*	9	/
*Veillonella*	11	/	*Veillonella dispar*	5	/
			*Veillonella parvula*	3	/
*Granulicatella*	9	/	*Granulicatella adiacens*	8	/
*Rothia*	3	/	*Rothia mucilaginosa*	3	/

## Discussion

The two typical cases reported in this article occurred in summer and autumn. Clinical studies have revealed an association between LD and climate; some 62% of cases occur in the summer and early autumn when precipitation increases (29), and 24% of cases are associated with travel (30). The first LD patient in this study presented with HAP, and she had high-risk factors for infection, such as a history of COPD and immunocompromising conditions (80 years old and steroid use). The second patient was a taxi driver who had worked in an air-conditioned environment for a long period, and had smoked for decades. These conditions are associated with a high risk of occupational exposure and infection with *Legionella* (31–33).

Interestingly, in our patients, the lower respiratory secretions appeared orange in color; a similar finding has been reported both for patients with Lp pneumonia and for patients with *Legionella longbeachae* pneumonia and may be another clue that should prompt specific testing (34–36). The exact mechanism through which this color appears is unknown, and it is presumed to be due to the utilization of tyrosine in the alveolar epithelial cell lining fluid by *Legionella* (37). In the second patient, despite significant improvement in respiratory symptoms, a generalized maculopapular rash and a high fever suddenly developed on approximately hospital day 12. LD-associated rash is relatively rare (38), and its pathogenesis is poorly understood. Its occurrence may be related to the presence of *Legionella* toxins (39, 40), host immune responses (41), or drug reactions (42). Rifampicin-associated delayed drug eruptions have also been reported (43); in this patient’s case, we also considered this as a possible cause of the rash, which subsided after appropriate treatment.

The etiological diagnosis of LD relies on microbiological laboratory tests. The culture of lower respiratory tract specimens, because it allows the isolation of strains for drug sensitivity testing and identification of all known strains of *Legionella* spp., is still the gold standard for diagnosis (44). However, it is rarely used in routine diagnosis because it requires stringent culture conditions, and the results can only be obtained after 3–5 days or even after 2 weeks (11).

Urine antigen tests (UATs) for *Legionella* are essential for the early diagnosis of LD (11) and have the advantages of convenience, timeliness and low cost. In Europe and the United States, diagnosis of LD is based on UATs in 70–80% of cases, and they have become the test of choice for diagnosing LD (45). However, most commercial UATs detect only Lp1 (46), which is isolated in approximately 80% of cases of Lp pneumonia (47, 48). In general, UATs are most sensitive to the Lp1 MAb 3/1 subtype, whereas their sensitivity in patients with Lp1 MAb 3/1-negative infection is approximately 40% (2). Although Lp1 is the major causative pathogen of LD (47, 48), regional differences are significant. The Lp1 subtype is responsible for 87.1% of community-acquired LDs in Japan (49), and for 80–95% of cases in the United States and Europe but for only approximately 50% of cases in Australia and New Zealand (48, 50, 51).

UATs are typically positive within 48–72 h of symptom onset and remain positive for weeks or months (2); positive results have, in fact, been reported nearly one year after an infectious episode (52). UATs may yield false negative or false positive results in the acute phase of infection. In addition, the sensitivity of UATs correlates with disease severity: they have 40–53% sensitivity for mild LD and 88–100% sensitivity for severe LD (53), likely due to differences in the antigen titers in urine samples. The use of highly concentrated urine samples can improve sensitivity, especially in cases involving mild disease, but samples are not routinely concentrated before testing (46, 54). A meta-analysis of 30 studies yielded a pooled sensitivity of 74.0% (95% CI, 68–81%) and a specificity of 99.1% (95% CI, 98.4–99.7%) for the UATs (55). As a result, in most cases in which the diagnosis is determined on the basis of UATs alone, epidemiological numbers may be underestimated compared with the actual incidence (44, 56).

PCR-based diagnosis of the presence of *Legionella* spp. is usually based on amplification of conserved regions of ribosomal RNA sequences. These regions are not specific and therefore can be used to detect any *Legionella* subspecies (57). In 35 studies that used respiratory samples, the summary sensitivity and specificity estimates of PCR for *Legionella* spp. were 97.4 and 98.6%, respectively (57). Compared with UATs, PCRs have better sensitivity and similar specificity. In the literature, patients with LDs that was acquired in the nosocomial setting or who are severely immunosuppressed are more likely to be infected by non-Lp1 strains (58, 59). For example, Head et al. reported that 36% of people living with HIV were coinfected with *Legionella* and that approximately one-third of LDs were caused by Lp, but none of these cases were caused by Lp1 (60). In such cases, the UATs might yield false negative results, and PCR might significantly improve the accuracy of diagnosis in these cases.

However, false negative results have been reported in some studies because of factors such as PCR inhibition, mismatch of primers and/or probes, the presence of *Legionella* targets in quantities below the limit of detection, and improper sample collection and handling (61). False positive results have also occurred due to cross-reaction of the *Legionella* species target (but not Lp) with *Stenotrophomonas maltophilia* (46). In addition, PCR kits are expensive, and the procedure requires specialized lab equipment and personnel. Although molecular tools such as specific PCR for *Legionella* spp. have been developed, they are rarely used in the clinic (62). In Europe, only 2% of 11,832 confirmed or suspected LD cases were identified by PCR (63).

mNGS enables the early detection of pathogenic microorganisms in specimens without hypothesis or bias, and it yields results that are not greatly influenced by previous administration of antibiotics (64). As a high-resolution and sensitive assay that covers the entire *Legionella* genus, including Lp and non-Lp, mNGS may circumvent the shortcomings of *Legionella* culture and UATs, and compensate for the inherent weaknesses of PCR in the diagnosis and surveillance of *Legionella* infection (65). In the literature coinfection with *Legionella* and other bacterial species, particularly *Streptococcus pneumoniae* and *Acinetobacter*, may occur (66). Tan et al. described six LD patients, all of whom had bacteremic coinfections (67). Other species that have been found to coinfect individuals infected with *Legionella* include *Pneumocystis jirovecii* (68), *Mycoplasma pneumoniae*, *Chlamydia pneumoniae*, *Chlamydia psittaci*, *Klebsiella pneumoniae*, and *Pneumocystis aeruginosa* (69). In cases such as these, mNGS can more comprehensively identify the coinfecting pathogens in a timely manner, and the mNGS results can be used to select the antibiotic mixture needed for the successful treatment of pneumonia.

We are currently in an era of rapid development of novel techniques such as mNGS. These novel techniques should be considered new tools that provided rapid molecular methods for the detection of pathogens in pneumonia patients. mNGS also has the potential to identify pathogens present in the environment, including *Legionella*, for example in water samples, and thus to play a crucial role in outbreak control (66). Identifying *Legionella* as the causative agent of infection is essential for disease treatment and outbreak prevention (70, 71).

As a rapid and unbiased assay for the presence of specific microorganisms, mNGS has unique advantages in the detection of difficult-to-culture pathogens (72), especially in resource-limited healthcare settings. However, mNGS also has several shortcomings compared with other traditional microbiological tests; these shortcomings include its higher cost, its analytical sensitivity, the need for a complex laboratory workflow, and its susceptibility to contamination (73). Although the current cost of mNGS is relatively high (approximately 480$ per sample in China), early precise pathogenic diagnosis, treatment and optimal patient management may help reduce overall medical expenditures (65). On the other hand, patients’ families often believe it is worthwhile to identify the pathogen early to improve patient prognosis. Nevertheless, the high cost of mNGS hinders its promotion and application in clinical practice. Therefore, targeted next-generation sequencing (tNGS) technology, which may be a good supplement for mNGS, is being developed and applied in China. Its sequencing volume is 1% that of mNGS, its cost is lower (approximately 164$ per sample in China), and it can detect hundreds of common DNA and RNA pathogens, thereby meeting the routine needs of clinical practice (74). However, if tNGS results are negative and the patient nevertheless has a highly suspicious infection, it is recommended that use of the mNGS test be considered to expand pathogen detection.

Given the vast amount of sequence information obtained through mNGS and the diversity of existing pathogen species, it can be extremely difficult to interpret mNGS reports correctly, and this increases the risk of false positive results (75). To date, there is a lack of internationally recognized standards for distinguishing whether pathogens identified by mNGS are truly pathogenic, colonized, or merely false positives. To address this issue, we combine the mNGS results with potentially useful information that may help identify the causative pathogen of lung infection; the other useful information includes the patient’s medical history, immune status, serum inflammation values, microbial culture results, lung imaging and many other types of clinical information. In addition, at least two associate chief physicians jointly interpret the mNGS results to increase the authenticity and reliability of the conclusions and to reduce false positive rates as much as possible.

UATs and PCR for *Legionella* have not yet been performed at our hospital; the two cases of severe LD discussed here were ultimately confirmed by mNGS of BALF. We believe that with reductions in sequencing costs and continuous improvement in interpretation standards in China, mNGS-based pathogenic diagnosis can be increasingly used to greatly improve the choice of antimicrobial drug regimens and the timeliness of clinical pathogen treatment, especially in challenging cases of complicated infectious disease (76).

## Conclusion

Overall mortality due to LD ranges from 4 to 18%, but it is close to 40% among immunocompromised patients and individuals requiring ICU admission (77–79). Therefore, early identification and diagnosis of LD is essential for effective treatment and accurate prognosis. Most clinical manifestations, laboratory findings, and radiographic features are non-specific. Orange-colored sputum and BALF appear to represent an important clues, but this needs to be explored in larger numbers of cases. The settings and incubation time required to culture *Legionella* are very strict, and not easy applied in clinical practice. UATs are convenient and fast, but mainly detect Lp1 and are not equally sensitive to all subtypes of *Legionella* (2), possibly resulting in underestimation of the actual incidence of the infection (44). PCR is more sensitive than UATs and can detect all *Legionella* subtypes, but it is technically demanding and is not widely used in the clinic (62). Therefore, mNGS could be considered for patients with severe pneumonia. In particular, causative pathogens cannot be identified by conventional detection methods (80) and patients may have coinfections. The use of mNGS can provide new diagnostic evidence that can be used to precisely guide precise antimicrobial therapy (81).

## Data Availability

The original contributions presented in the study are included in the article/supplementary material, further inquiries can be directed to the corresponding author.
